# Molecular mechanism of LncRNA MALAT1 in regulating hepatocellular carcinoma progression via the miR-383-5p/PRKAG1 axis and its role in the tumor immune microenvironment

**DOI:** 10.3389/fonc.2025.1613596

**Published:** 2025-09-01

**Authors:** Rui Zhang, Mingze Li, Shan Lu, Anni Wang, Shujun Zhang

**Affiliations:** ^1^ Department of Pathology, The Fourth Affiliated Hospital of Harbin Medical University, Harbin, Heilongjiang, China; ^2^ Department of Neurosurgery, The First Affiliated Hospital of Harbin Medical University, Harbin, Heilongjiang, China

**Keywords:** hepatocellular carcinoma, MALAT1, PRKAG1, miR-383-5p, tumor immune microenvironment

## Abstract

**Background:**

Hepatocellular carcinoma (HCC) is a highly prevalent malignant tumor worldwide, and its development is closely associated with dysregulated non-coding RNA expression. The long non-coding RNA MALAT1 is overexpressed in multiple cancers, but its precise mechanistic role and downstream regulatory network in HCC remain incompletely understood. Additionally, PRKAG1, a regulatory subunit of AMPK, has an unclear function in tumor progression. This study aimed to investigate the role and clinical significance of the MALAT1-PRKAG1 axis in HCC pathogenesis.

**Methods:**

A multi-omics approach was employed to systematically dissect the mechanism of the MALAT1-PRKAG1 axis in HCC. Bioinformatics analysis using GEPIA2 and TCGA databases revealed that MALAT1 and PRKAG1 were significantly upregulated in HCC and correlated with poor prognosis. Cellular experiments demonstrated that knockdown of MALAT1 or PRKAG1 suppressed HCC cell proliferation, migration, and invasion. Mechanistic studies indicated that MALAT1 upregulates PRKAG1 expression by competitively binding miR-383-5p. Further analyses showed that PRKAG1 remodels the tumor immune microenvironment by modulating immune cell infiltration (CIBERSORT analysis) and intercellular communication (single-cell RNA sequencing), while also activating key pathways such as the cell cycle and DNA repair (GO/KEGG enrichment analysis).

**Results:**

This study uncovered the critical role of the MALAT1-PRKAG1 regulatory axis in HCC. MALAT1 was significantly overexpressed in HCC tissues and cell lines, and its expression was associated with poor patient prognosis. Knockdown of MALAT1 markedly inhibited HCC cell proliferation, migration, and invasion. PRKAG1, as a downstream target of MALAT1, was also highly expressed in HCC and correlated with tumor stage and adverse outcomes. Mechanistically, MALAT1 competitively binds miR-383-5p to relieve its suppression of PRKAG1, thereby activating the P53 and AKT signaling pathways. Additionally, PRKAG1 modulated immune cell infiltration (particularly CD4+ T cells and M0 macrophages) and promoted intercellular communication via the MIF signaling network. Multi-omics analysis revealed that PRKAG1-associated genes were primarily enriched in critical pathways, including the cell cycle and DNA repair, collectively driving HCC progression.

**Conclusion:**

This study elucidates the oncogenic role of the MALAT1/miR-383-5p/PRKAG1 axis in HCC, demonstrating that PRKAG1 promotes tumor progression by regulating cell proliferation, the immune microenvironment, and key signaling pathways. These findings provide potential novel targets for HCC prognosis assessment and targeted therapy.

## 1 Introduction

Hepatocellular carcinoma (HCC) ranks among the most prevalent malignancies worldwide, with particularly high incidence and mortality rates in China, posing a significant public health burden (1). The disease is characterized by insidious onset, rapid progression, and frequent recurrence, leading to generally poor clinical outcomes (2). Therefore, elucidating the molecular mechanisms underlying HCC pathogenesis and identifying novel therapeutic targets and prognostic biomarkers are crucial for improving patient survival and quality of life.

In recent years, long non-coding RNAs (lncRNAs) have emerged as key regulators in tumor biology. Among them, metastasis-associated lung adenocarcinoma transcript 1 (MALAT1), a well-studied lncRNA, exhibits aberrant expression in multiple malignancies and is closely associated with tumor growth, metastasis, and prognosis (3). In HCC, MALAT1 is significantly upregulated, and its expression correlates with clinicopathological features and patient outcomes. Beyond its roles in RNA splicing and transcriptional regulation, MALAT1 modulates various cancer-related biological processes, including proliferation, migration, and invasion (4).

MicroRNAs (miRNAs), a class of short non-coding RNAs, play pivotal roles in tumorigenesis by regulating mRNA expression (5). Notably, miR-383-5p has been demonstrated to suppress tumor cell proliferation, migration, and invasion, highlighting its potential as both a prognostic marker and therapeutic target in multiple cancers (6). However, the crosstalk between MALAT1 and miR-383-5p, as well as their regulatory mechanisms in HCC, remains unclear.

Additionally, protein kinase AMP-activated non-catalytic subunit γ1 (PRKAG1), a critical component of the AMPK signaling pathway, participates in tumorigenesis by regulating cellular energy metabolism and stress responses (7). Dysregulation of AMPK pathway components has been linked to the progression of various cancers (8), yet the precise mechanistic role of PRKAG1 in tumor biology warrants further investigation.

The tumor immune microenvironment (TIME) plays a decisive role in HCC development and therapeutic resistance (9). Studies indicate that the composition and functional states of immune cells within the TIME, along with their interactions with tumor cells, collectively influence HCC progression. In particular, the infiltration of immunosuppressive cells—such as tumor-associated macrophages (TAMs), regulatory T cells (Tregs), and myeloid-derived suppressor cells (MDSCs)—often contributes to immune evasion and tumor aggressiveness (10). Emerging evidence suggests that mRNAs can remodel the immunosuppressive properties of TIME by mediating intercellular communication between tumor and immune cells. For instance, certain mRNAs facilitate crosstalk via exosomal pathways or modulate cytokine secretion to alter immune microenvironments (11). Nevertheless, the involvement of the PRKAG1 axis in TIME regulation, particularly its impact on immune cell functionality and underlying mechanisms, remains poorly understood.

Based on these observations, this study aims to investigate the regulatory axis of MALAT1/miR-383-5p/PRKAG1 in HCC. We hypothesize that MALAT1 may modulate PRKAG1 activity and downstream signaling pathways by sponging miR-383-5p, thereby driving HCC pathogenesis. A comprehensive dissection of this axis could provide novel insights for HCC diagnosis and treatment, offering both theoretical significance and potential clinical applications.

## 2 Materials and methods

### 2.1 Gene expression analysis

We retrieved RNA and protein expression profiles of PRKAG1 across human tissues, including its expression in various immune cell types, from the Human Protein Atlas (HPA) database (https://www.proteinatlas.org/) (12). Based on these data, we constructed a comprehensive PRKAG1 mRNA expression atlas.

To investigate differential PRKAG1 expression between tumor and non-tumor tissues, we utilized the “Gene DE” module in TIMER 2.0 (http://timer.cistrome.org/) (13). Statistical comparisons were performed to assess PRKAG1 expression levels across 33 cancer types, including: Adrenocortical carcinoma (ACC), Bladder urothelial carcinoma (BLCA), Breast invasive carcinoma (BRCA), Cervical squamous cell carcinoma and endocervical adenocarcinoma (CESC), Cholangiocarcinoma (CHOL), Colon adenocarcinoma (COAD), Diffuse large B-cell lymphoma (DLBC), Esophageal carcinoma (ESCA), Glioblastoma multiforme (GBM), Head and neck squamous cell carcinoma (HNSC), Kidney chromophobe (KICH), Kidney renal clear cell carcinoma (KIRC), Kidney renal papillary cell carcinoma (KIRP), Acute myeloid leukemia (LAML), Brain lower-grade glioma (LGG), Liver hepatocellular carcinoma (LIHC), Lung adenocarcinoma (LUAD), Lung squamous cell carcinoma (LUSC), Mesothelioma (MESO), Ovarian serous cystadenocarcinoma (OV), Pancreatic adenocarcinoma (PAAD), Pheochromocytoma and paraganglioma (PCPG), Prostate adenocarcinoma (PRAD), Rectum adenocarcinoma (READ), Sarcoma (SARC), Skin cutaneous melanoma (SKCM), Stomach adenocarcinoma (STAD), Testicular germ cell tumors (TGCT), Thyroid carcinoma (THCA), Thymoma (THYM), Uterine corpus endometrial carcinoma (UCEC), Uterine carcinosarcoma (UCS), Uveal melanoma (UVM).

### 2.2 Survival and prognostic analysis

To comprehensively evaluate the association between PRKAG1 expression and patient survival across multiple cancers, we employed the “Survival Analysis” module in GEPIA 2 (http://gepia2.cancer-pku.cn/) (14), assessing overall survival (OS) and disease-free survival (DFS). Kaplan-Meier (K-M) curves were generated to compare survival differences between high- and low-expression groups (defined as the 75th and 25th percentiles, respectively) using data from The Cancer Genome Atlas (TCGA).

Clinical-pathological characteristics (age, sex, TNM stage, etc.) and OS data from HCC patients were collected. Univariate and multivariate Cox regression analyses were performed to identify prognostic variables (P < 0.05). Patients were stratified into high- and low-expression groups based on median PRKAG1 expression, and Kaplan-Meier/log-rank tests assessed prognostic significance. Variables with P < 0.1 in univariate analysis, along with PRKAG1 expression, were included in multivariate Cox models to validate PRKAG1 as an independent prognostic factor. Forest plots were generated using the R package forestplot (v4.2.0) to visualize hazard ratios (HRs) and 95% confidence intervals, with HR = 1 as the reference. All analyses were conducted using survival and survminer packages (significance threshold: α = 0.05).

### 2.3 Immune cell infiltration analysis

Using TIMER 2’s “Immune” module (http://timer.cistrome.org/), we analyzed PRKAG1 expression across cancers and its correlation with immune infiltration scores (EPIC, TIMER, TIDE, MCPCOUNTER). This evaluated PRKAG1’s role in immune evasion and its association with immune cell infiltration (CD4+ T cells, CD8+ T cells, B cells, cancer-associated fibroblasts [CAFs]) in TCGA-HCC samples. Samples were divided into high- and low-expression groups based on PRKAG1 levels. CIBERSORT (LM22 signature matrix) and ESTIMATE algorithms quantified immune infiltration (1000 permutations; significant results retained at P < 0.05). Wilcoxon rank-sum tests compared 22 immune cell subsets between groups, with Benjamini-Hochberg correction (FDR < 0.05). Boxplots and heatmaps visualized significant differences. Analyses used normalized expression matrices (TPM/FPKM).

### 2.4 Gene enrichment analysis

The STRING database (v11.0b; https://string-db.org/) (15) constructed a PRKAG1 co-expression network with parameters:

Interaction sources: Co-expression;

Network edges: Evidence;

Maximum interactors: 50;

Minimum interaction score: Low confidence (0.150).

GEPIA 2’s “Similar Genes Detection” identified 50 genes with expression patterns most correlated to PRKAG1 in TCGA. Gene Ontology (GO) enrichment was performed using R’s clusterProfiler (v4.3.3) (16). Gene-pair correlations were analyzed via GEPIA 2’s “Correlation Analysis.”

### 2.5 Gene set variation analysis

Using MSigDB’s c2.cp.kegg.v7.4.symbols.gmt as reference, single-sample GSEA (ssGSEA) via the R package GSVA quantified KEGG pathway activity in high/low PRKAG1 groups. Wilcoxon tests identified differentially enriched pathways (|log2FC| > 0.1, P_adjust_ < 0.05). Top 10 enriched pathways per group were visualized using ggplot2.Differential expression analysis (high- vs. low-risk groups) was performed with DESeq2, followed by GSEA (clusterProfiler). Top 10 pathways (FDR < 0.25, P < 0.01) ranked by normalized enrichment score (NES) were plotted (enrichplot).

### 2.6 Methylation analysis of PRKAG1-associated sites

Methylation 450K/850K array and gene expression data were obtained from Xena (17). Methylation sites in PRKAG1 regulatory regions (promoter ± 2 kb, 5′UTR, first exon) were analyzed. Pearson correlation identified sites with |r| > 0.3 and FDR < 0.05 linked to PRKAG1 expression.

### 2.7 Single-cell RNA sequencing-based PRKAG1 expression profiling and cell-cell communication analysis

Single-cell RNA-seq (scRNA-seq) data from HCC and adjacent non-tumor tissues were obtained from public databases. Quality control was performed using Seurat (v4.3.0), retaining cells expressing 200–6,000 genes with mitochondrial gene content <20%. Data were log-normalized, and batch effects were corrected using Harmony (v0.1.1). Principal component analysis (PCA) and uniform manifold approximation and projection (UMAP) were applied for dimensionality reduction and visualization. Major cell types—including hepatocytes (ALB+), monocytes (CD14+), myeloid cells (CD68+), T cells (CD3D+), and T/NK cells (NKG7+)—were annotated based on canonical markers.

Differential PRKAG1 expression between tumor and non-tumor tissues was assessed using the Wilcoxon rank-sum test (|log2FC| > 0.25, Bonferroni-adjusted P < 0.05), revealing significant variations across these cell types. To investigate altered cell-cell communication, we employed CellChat (v1.6.1) to analyze ligand-receptor interaction networks and PRKAG1-associated signaling pathways in differentially expressed cell populations. All analyses were conducted in R 4.2.0, with visualizations generated using ggplot2 (v3.4.2).

### 2.8 Evolutionary conservation analysis

The evolutionary conservation of PRKAG1 was evaluated using the UCSC Genome Browser (http://www.genome.ucsc.edu/cgi-bin/hgTracks) (18). After selecting appropriate vertebrate genome assemblies, we mapped PRKAG1 genomic coordinates and analyzed sequence alignment and annotation features across species.

### 2.9 Cell culture

HCC cell lines (MHCC97H, HUH7, SK-Hep-1, HEPA1-6) and the immortalized normal hepatocyte line (LO2) were provided by the Fourth Affiliated Hospital of Harbin Medical University. Cells were maintained in DMEM supplemented with 10% fetal bovine serum (FBS) at 37°C under 5% CO_2_. Subculturing or experiments were performed at 50–80% confluency. All cell lines were confirmed mycoplasma-free prior to use.

### 2.10 Lentiviral vector construction and transduction

shRNA targeting human PRKAG1 was designed and cloned into the pLV3 vector downstream of the U6 promoter. The construct also contained a CopGFP reporter gene and a puromycin resistance (Puro) marker, yielding the final lentiviral vector pLV3-U6-PRKAG1(human)-shRNA-CopGFP-Puro (Miaoling Biotechnology, Wuhan).Lentiviral particles were packaged in HEK 293T cells, concentrated, and transduced into MHCC97H and HUH7 cells. Stable PRKAG1 knockdown or overexpression cell lines were selected using puromycin.

### 2.11 siRNA design and transfection

Short interfering RNA (siRNA) targeting MALAT1 (siMALAT1) and a negative control siRNA (siNC), as well as miR-383-5p inhibitors, were synthesized by Ribobio (Guangzhou, China). For transfection, 2×10^6^ cells were treated with 4 μg of miR-383-5p inhibitor, the corresponding negative control (NC) primer, or 8 μg of siRNA using Lipofectamine 3000 (Invitrogen). Transfected cells were incubated for 48 hours before proceeding with subsequent experiments.

### 2.12 Quantitative real-time PCR

Total RNA was extracted with TRIzol reagent (Takara, Dalian) and reverse-transcribed using a PrimeScript RT kit (Takara). Amplification was performed with SYBR Green Master Mix (Takara) on a QuantStudio Real-Time PCR System, following manufacturer protocols. Relative expression was calculated via the 2-ΔΔCt method, with GAPDH as the internal control. Technical triplicates were included for all assays. The primer sequences used for qRT-PCR are listed in **Table 1**.

**Table 1:** Primer table for gene sequences.

Primer type	Sequence
MALAT1 forward	5′-AAAGCAAGGTCTCCCCACAAG-3′
MALAT1 reverse	5′-GGTCTGTGCTAGATCAAAAGGC-3′
PRKAG1 forward	5′-TCGGAACAAGATCCACAGGCTG-3′
PRKAG1 reverse	5′-CTTCTCTCCACCTGTGAGCACC-3′
si-MALAT1	5′-GCAAAUGAAAGCUACCAAUTT-3′
sh-PRKAG1	5′-GACTAATTCAAGAGATTAGTC-3′
miR-383-5p forward	5′-GCGCGAGATCAGAAGGTGATT-3′
miR-383-5p reverse	5′-AGTGCAGGGTCCGAGGTATT-3′
miR-383-5p inhibitor sense	5′-AGCCACAAUCACCUUCUGAUCU-3′
MIF forward	5’-AGAACCGCTCCTACAGCAAGCT-3’
MIF reverse	5’-GGAGTTGTTCCAGCCCACATTG-3’
CD74 forward	5’-AAGCCTGTGAGCAAGATGCGCA-3’
CD74 reverse	5’-AGCAGGTGCATCACATGGTCCT-3’
CXCR4 forward	5’-CTCCTCTTTGTCATCACGCTTCC-3’
CXCR4 reverse	5’-GGATGAGGACACTGCTGTAGAG-3’
GAPDH forward	5′-CTGGGCTACACTGAGCACC-3′
GAPDH reverse	5′-AAGTGGTCGTTGAGGGCAATG-3′

### 2.13 Western blot analysis

Total proteins were extracted from cell lines using RIPA lysis buffer (P0013B, Beyotime, USA) and quantified with a BCA protein assay kit (P0011, Beyotime). Equal amounts of protein (10 μg) were separated by SDS-PAGE (P0690, Beyotime) and subsequently transferred to PVDF membranes (P0965-100pcs, Beyotime). After blocking the membranes with 5% non-fat milk for 2 hours, the membranes were incubated with the following primary antibodies at 4°C overnight: PRKAG1 (1:1500, 10290-1-AP, Proteintech), MIF (1:2000, cy6829, Abways), CD74 (1:2000, cy6704, Abways), CXCR4 (1:1000, cy5380, Abways), Akt (1:2000, ab8805, Abcam), P53 (1:2000, ab50887, Abcam), α-Tubulin (1:5000, 11224-1-AP, Proteintech), and β-actin (1:20000, 66009-1-Ig, Proteintech). Subsequently, the membranes were incubated with corresponding HRP-conjugated secondary antibodies at room temperature for 1 hour: goat anti-mouse IgG (1:5000, ZB-2305, Zhongshan Golden Bridge) and goat anti-rabbit IgG (1:5000, ZB-2301, Zhongshan Golden Bridge). Protein bands were developed using an enhanced chemiluminescence (ECL) substrate (BL520B, Biosharp, China) and imaged with the Image Lab 3.0 system (Bio-Rad, USA).

### 2.14 Cell proliferation assay (CCK-8)

Cells were seeded into 96-well plates at a density of 3×10³ cells/well (100 μL/well, 5 replicate wells per group). At 0, 24, 48, 72, and 96 hours, 10 μL of CCK-8 solution (Dojindo, Japan) was added to each well, followed by incubation at 37°C for 4 hours. The absorbance at 450 nm was measured using a microplate reader (Tecan, Switzerland).

### 2.15 Wound healing assay

Cells were seeded into 6-well plates (1×10^5^ cells/well) and cultured until reaching 90% confluence. Linear scratches (approximately 0.5 mm in width) were created using a sterile 200 μL pipette tip. After washing with PBS to remove cell debris, cells were cultured in serum-free medium. The wound healing process was observed at 0, 24, and 48 hours using an inverted microscope (Leica, Germany). The migration distance was quantified using ImageJ software (NIH, USA), and the wound healing rate was calculated as

follows:


$$\text{Wound healing rate (\%) = }\left(\frac{Wound width at 24h/48h - Wound width at 0h}{Wound width at 0h}\right)\;\times \;100\%$$

### 2.16 Colony formation assay

Cells were seeded into 6-well plates at a density of 1,000 cells/well and cultured for 14 days. After gentle washing with PBS, colonies were fixed with 4% paraformaldehyde for 2 hours and then stained with 0.1% crystal violet for 30 minutes. Images of colonies were captured using an inverted microscope (Leica, Germany), and the number of colonies was manually counted in 3 randomly selected fields per well.

### 2.17 Transwell invasion assay

The upper chamber of Transwell inserts (Corning, USA) was coated with 50 μL of Matrigel matrix (BD Biosciences, USA). Cells were resuspended in serum-free DMEM at a density of 1×10^5^ cells/mL, and 100 μL of the cell suspension was added to each upper chamber. The lower chamber was filled with 600 μL of DMEM containing 10% FBS as a chemoattractant. After 48 hours of incubation, cells that invaded through the Matrigel were fixed with 4% paraformaldehyde for 2 hours and stained with 0.1% crystal violet for 30 minutes. After washing and drying the membranes, images were captured using a light microscope (Nikon, Japan). The number of invasive cells was manually counted in 3 randomly selected fields per membrane.

### 2.18 *In vivo* experiments

Specific pathogen-free (SPF) male BALB/c mice (6–8 weeks old, 20–25 g, n=5 per group) were randomly divided into the Hepa1-6-shPRKAG1 group and the shNC group, and housed under SPF conditions. Hepa1–6 cells in the logarithmic growth phase were adjusted to 1×10^6^ cells/mL with PBS, and 50 μL of the cell suspension was injected into the left lobe of the liver through an incision under the left costal margin of the mice at a rate of 10 μL/min. After suturing, mice were housed individually. Tumor formation was monitored weekly by *in vivo* imaging (after injection of luciferin substrate). After 4 weeks, mice were euthanized, and tumor volume was measured during dissection using the formula: V = length × width²/2. Tumor tissues and adjacent non-tumor tissues were fixed with 4% paraformaldehyde or frozen in liquid nitrogen for subsequent analyses. All experiments were conducted in accordance with animal ethics guidelines (Approval No.: 2025-DWSYLLCZ-85).

### 2.19 HE staining and immunohistochemistry

For HE staining, 4–5 μm sections were prepared. After dewaxing in xylene (5 minutes × 3 times), sections were hydrated through a graded ethanol series (100% to 80%) and rinsed with distilled water for 5 minutes. Sections were stained with Harris hematoxylin at room temperature for 5–10 minutes, rinsed with tap water to develop color, differentiated with 1% hydrochloric acid-70% ethanol for 3–5 seconds, and then rinsed with tap water for 10 minutes to blue the nuclei. Subsequently, sections were stained with 0.5% eosin for 2–5 minutes, briefly rinsed with distilled water, dehydrated through a graded ethanol series, cleared in xylene, and finally mounted with neutral balsam. Stained sections were observed under a light microscope (model BX53, Olympus), and images were captured at 40× magnification. For cultured cells on coverslips, the procedure was similar: cells were first rinsed with PBS, fixed with 4% paraformaldehyde, followed by staining, dehydration, and observation.

For immunohistochemical staining, paraffin sections were dewaxed in xylene and hydrated through a graded ethanol series. Antigen retrieval was performed by microwaving sections in 0.01 M citrate buffer (pH 6.0) for 15 minutes, followed by cooling at room temperature for 20 minutes. Endogenous peroxidase activity was blocked by incubating sections with 3% hydrogen peroxide at room temperature for 10 minutes, and non-specific binding was blocked with 5% bovine serum albumin for 30 minutes. Sections were incubated with primary antibodies against PRKAG1 (1:200, 10290-1-AP, Proteintech), MIF (1:200, cy6829, Abways), CD74 (1:200, cy6704, Abways), and CXCR4 (1:200, cy5380, Abways) at 4°C overnight. After washing with PBST, sections were incubated with HRP-conjugated secondary antibody (1:500, AB0153, Abways) at 37°C for 1 hour. After diaminobenzidine (DAB) development, sections were counterstained with hematoxylin for 3 minutes, dehydrated, cleared, and mounted. Images were captured using an Olympus BX53 microscope. For negative controls, primary antibodies were replaced with PBS.

### 2.20 Statistical analysis

All experimental data are presented as **mean ± standard error of the mean (SEM)**. Statistical analyses and data visualization were performed using R software (version 4.3.3) and IBM SPSS Statistics (version 27.0). Comparisons between two groups were analyzed using unpaired Student’s t-test, and comparisons among multiple groups were analyzed using one-way analysis of variance (ANOVA). Pearson correlation analysis was used to evaluate associations between variables. Prognostic significance was analyzed using Kaplan-Meier survival curves, and survival differences were assessed using the log-rank test. A two-tailed P-value < 0.05 was considered statistically significant.

## 3 Results

### 3.1 High expression of MALAT1 in hepatocellular carcinoma tissues and cell lines

**Figure 1:**
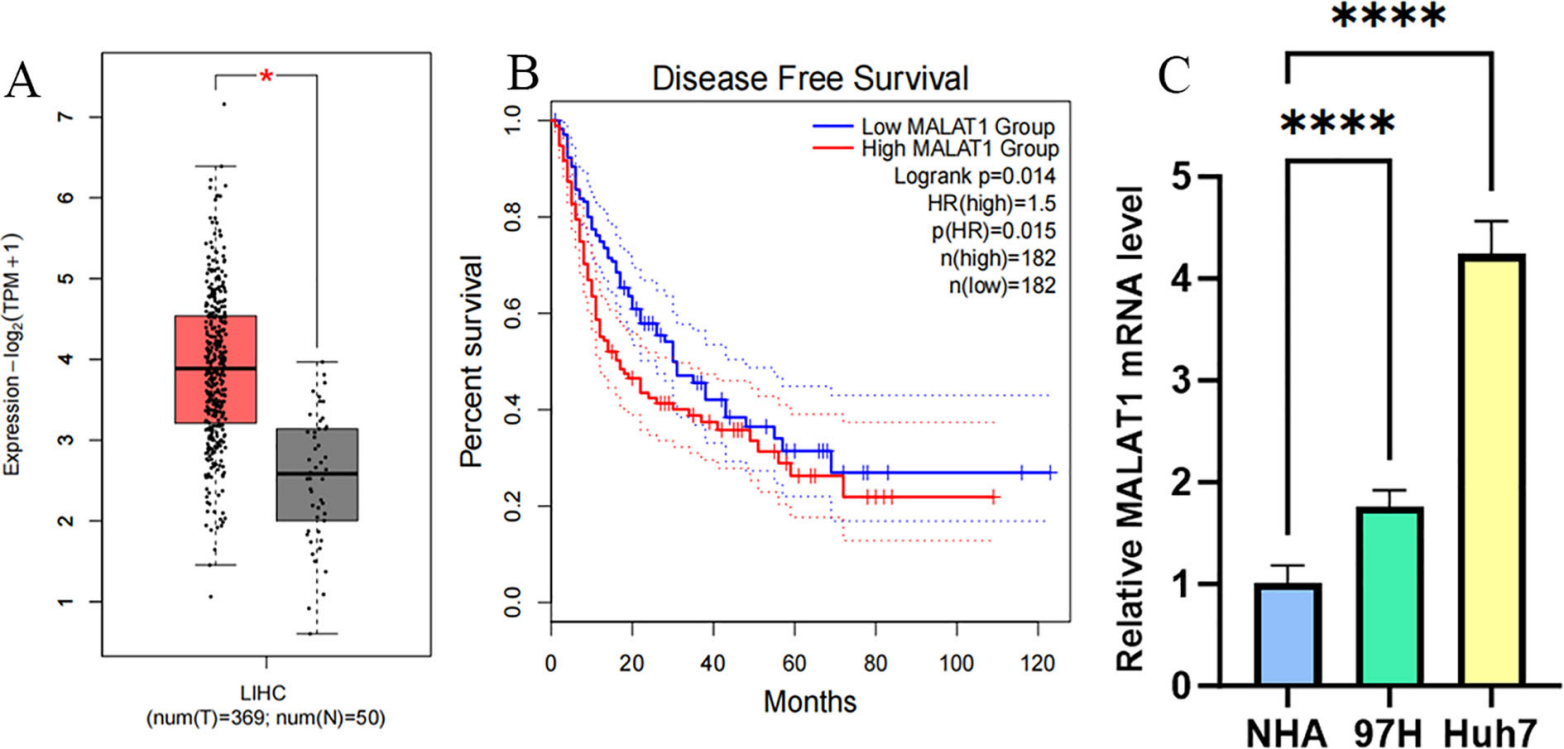
MALAT1 Expression in Liver Cancer Samples/Cell Lines. **(A)** GEPIA2 database was used to measure MALAT1 expression in samples obtained from liver cancer patient samples (n = 369) and healthy volunteers (n = 50). **(B)** Survival curves were constructed using GEPIA2 to analyze the correlation between MALAT1 expression and Disease Free Survival in patients with different TCGA tumor types. The 95% confidence intervals for overall survival in the high MALAT1 group and low MALAT1 group are represented by red and blue dashed lines, respectively. **(C)** Q-PCR was used to measure MALAT1 expression in MHCC97H/Huh-7 cell lines and healthy human hepatocytes. *p < 0.05; ****p < 0.0001.

Analysis of TCGA data from 369 hepatocellular carcinoma (HCC) patients and 50 healthy controls using the GEPIA2 database revealed that the expression level of MALAT1 in tumor tissues was significantly higher than that in normal tissues (**Figure 1A**). Kaplan-Meier survival analysis showed that high MALAT1 expression in TCGA HCC tissues was negatively correlated with disease-free survival (DFS) (**Figure 1B**), with a significantly increased risk of recurrence in the high-expression group. Additionally, detection in human HCC cell lines demonstrated that MALAT1 expression levels in MHCC97H and Huh7 cells were significantly higher than those in normal hepatocyte lines (**Figure 1C**).

### 3.2 MALAT1 promotes proliferation, migration, and invasion of hepatocellular carcinoma cells

**Figure 2:**
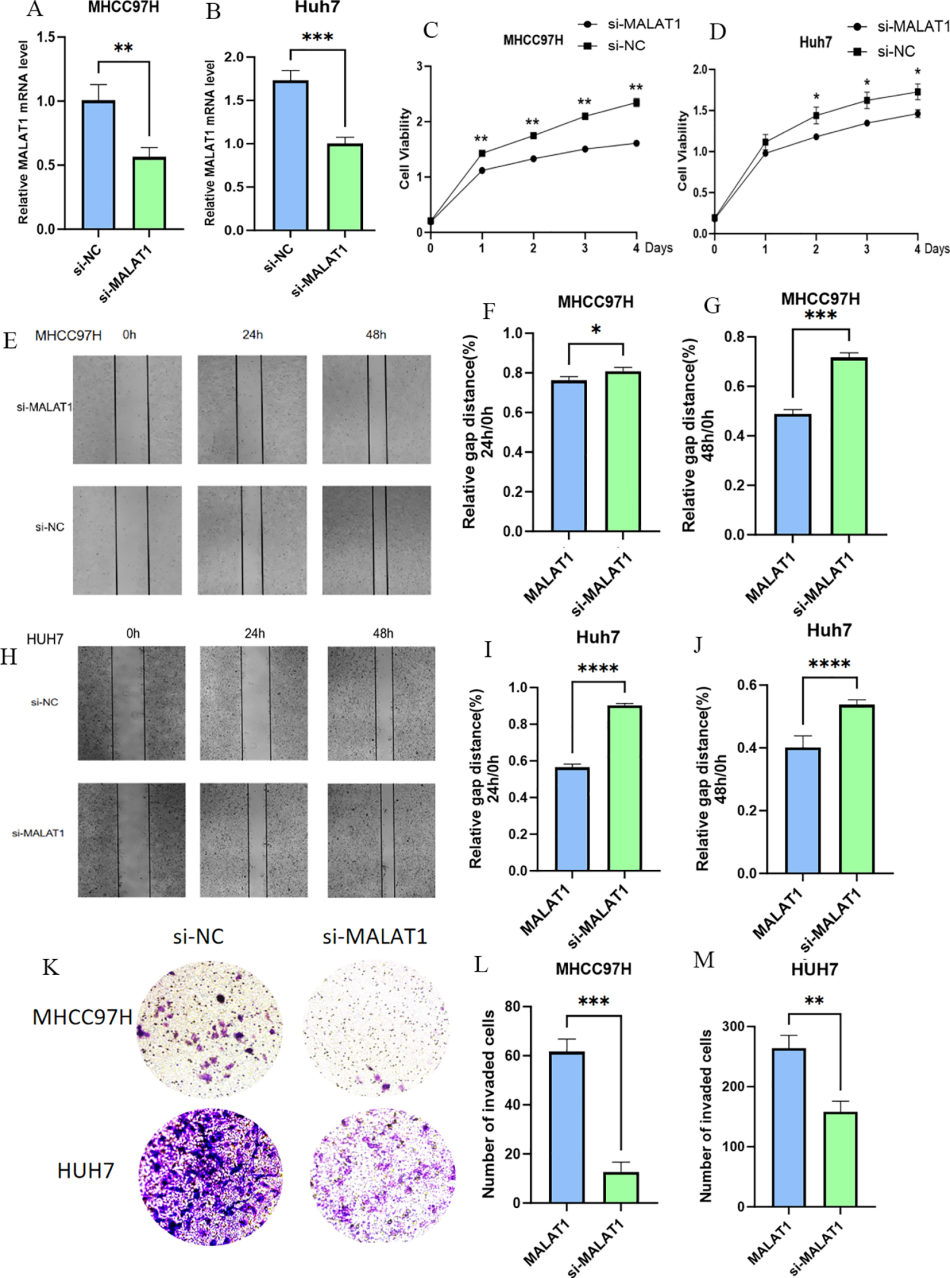
The Role of MALAT1 in Liver Cancer Cells. **(A, B)** Q-PCR analysis of MALAT1 expression in MHCC97H and Huh-7 cells after transfection with siMALAT1 or siNC. **(C, D)** CCK-8 assay showing proliferation rates at 24, 48, 72, and 96 hours. **(E-J)** Wound healing assay at 24 and 48 hours post-transfection. **(K-M)** Transwell assay for cell invasion. Data are mean ± SD; *p < 0.05; **p < 0.01; ***p < 0.001; ****p < 0.0001.

To investigate the biological function of MALAT1 in HCC, loss-of-function experiments were performed in MHCC97H and Huh-7 cell lines. After effective knockdown of MALAT1 via siRNA transfection (**Figures 2A, B**), CCK-8 assays showed that the proliferation rate of the siMALAT1 group was significantly reduced at 48–96 hours compared to the negative control group (siNC) (**Figures 2C, D**). Wound healing assays confirmed that MALAT1 knockdown significantly inhibited the migration ability of HCC cells (**Figures 2E–J**), while Matrigel invasion assays further demonstrated that MALAT1 enhanced the invasive potential of HCC cells (**Figures 2K–M**). These results suggest that MALAT1 plays a critical role in HCC progression by regulating the proliferation, migration, and invasion of HCC cells.

### 3.3 MALAT1 regulates PRKAG1 expression: identification of an independent prognostic predictor in hepatocellular carcinoma

**Figure 3:**
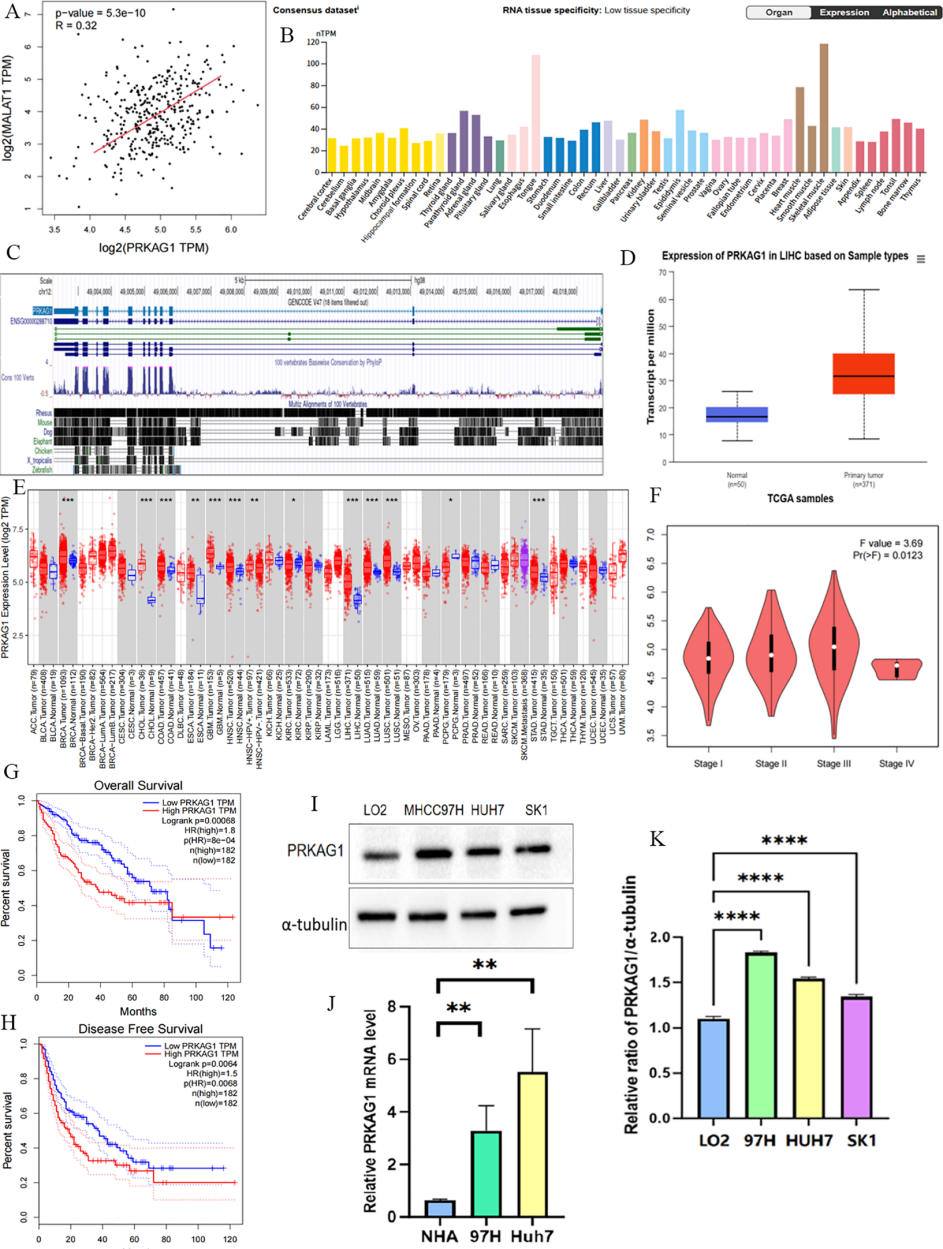
NA Expression Profile of PRKAG1 in Human Organs and Tissues. **(A)** TCGA database analysis of the correlation between MALAT1 expression and PRKAG1 expression in liver cancer patient samples. **(B)** Summary of PRKAG1 mRNA expression in human organs and tissues. **(C)** Visualization of PRKAG1 gene conservation analysis across vertebrates using the UCSC Genome Browser. **(D)** Visualization of PRKAG1 expression status in different tumor types using TIMER 2. **(E)** UALCAN analysis of PRKAG1 expression in liver cancer patient samples versus healthy volunteer samples. **(F)** GEPIA2 analysis of the correlation between PRKAG1 expression and clinical stages. **(G, H)** Kaplan-Meier survival curve analysis of the correlation between PRKAG1 expression and overall survival in patients with different TCGA tumor types. **(I, J)** Western blot analysis of PRKAG1 expression in liver cancer cells versus normal liver cells. **(K)** Q-PCR analysis of PRKAG1 expression in liver cancer cells versus normal liver cells. *p < 0.05; **p < 0.01; ***p < 0.001; ****p < 0.0001.

To clarify the mechanism of MALAT1 in HCC, we screened target genes associated with MALAT1 that are highly expressed in HCC tissues and positively correlated with poor prognosis, ultimately identifying PRKAG1 as the core research object. Data showed a significant correlation between PRKAG1 expression and MALAT1 (**Figure 3A**), prompting further exploration of the biological significance of PRKAG1 in HCC. Analysis of the FANTOM5 (Functional Annotation of Mammalian Genomes) dataset revealed that PRKAG1 is enriched in brain, esophagus, liver, and skeletal muscle tissues (**Figure 3B**), with low tissue specificity and high conservation in vertebrates (**Figure 3C**). Notably, the expression pattern of PRKAG1 in tumor tissues was similar to its distribution in normal tissues, also showing low tumor specificity.

Comparative analysis indicated that PRKAG1 mRNA was significantly upregulated in various tumor tissues, including breast invasive carcinoma (BRCA), cholangiocarcinoma (CHOL), colon adenocarcinoma (COAD), glioblastoma multiforme (GBM), head and neck squamous cell carcinoma (HNSC), liver hepatocellular carcinoma (LIHC), lung adenocarcinoma (LUAD), lung squamous cell carcinoma (LUSC), and stomach adenocarcinoma (STAD) (all P<0.001) (**Figure 3E**). TCGA data further confirmed this finding and revealed that PRKAG1 expression was associated with HCC pathological stages (**Figures 3D, F**). Kaplan-Meier survival analysis showed that high PRKAG1 expression in TCGA HCC tissues was negatively correlated with overall survival (OS) and recurrence-free survival (RFS) (both P<0.001), with significantly poorer OS and DFS in the high-expression group compared to the low-expression group (**Figures 3G, H**). Western blot analysis showed that PRKAG1 expression in HCC cell lines was significantly higher than that in normal hepatocyte lines (**Figures 3I, J**); qPCR results confirmed that PRKAG1 mRNA expression levels in MHCC97H and Huh-7 cells were significantly higher than those in normal human hepatocytes (**Figure 3K**). These lines of evidence indicate that PRKAG1 is a downstream target of MALAT1 and may serve as an independent prognostic marker for HCC, with its overexpression associated with adverse clinical outcomes.

**Figure 4:**
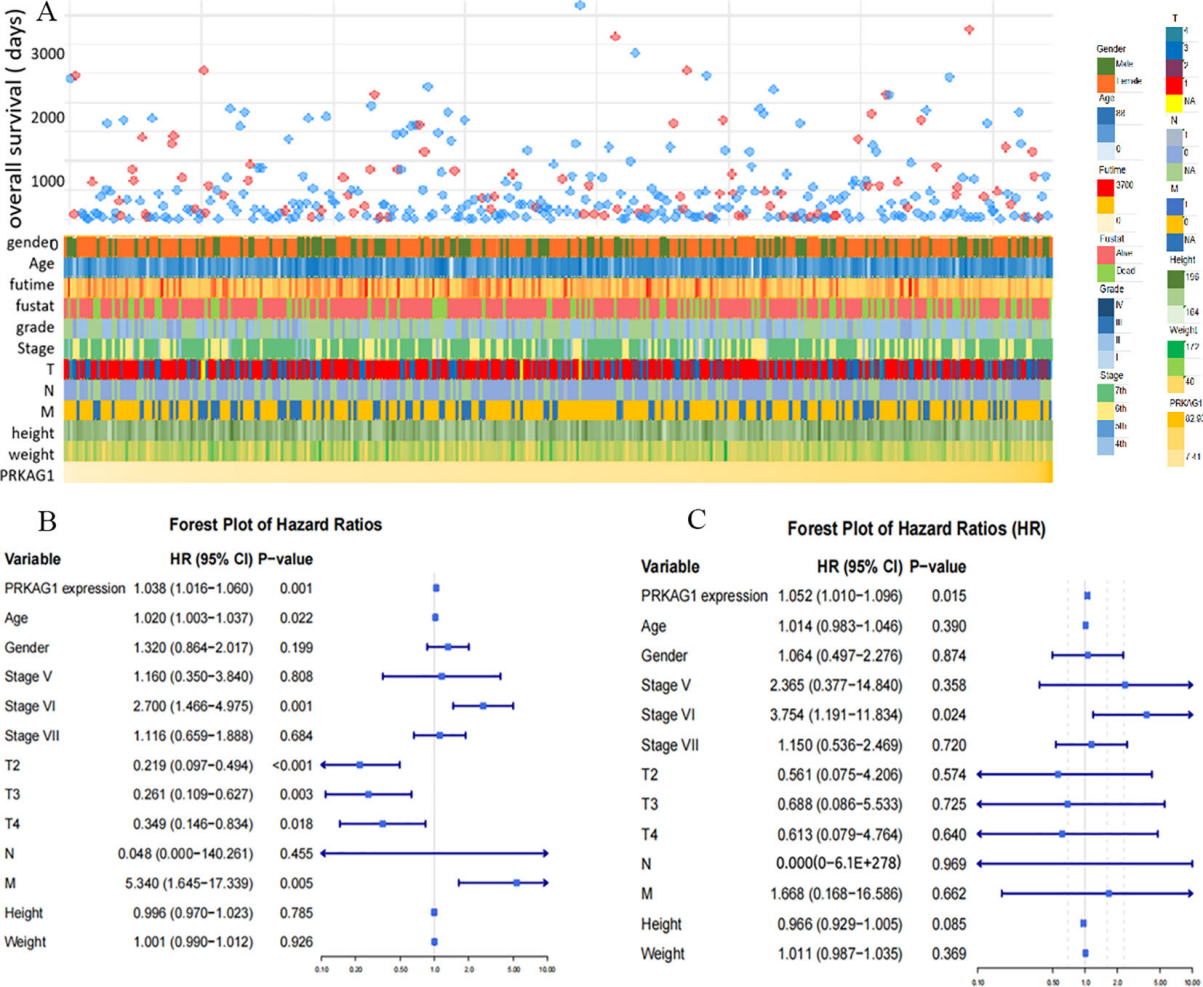
PRKAG1 May Be an Independent Prognostic Factor for HCC. **(A)** TCGA database analysis of the correlation between PRKAG1 and clinicopathological characteristics of patients. **(B)** Cox regression analysis was utilized to explore the correlation between PRKAG1 and patient prognosis.

Further analysis revealed that patients with different PRKAG1 expression levels had significantly different clinicopathological characteristics. TCGA data showed variations in the distribution of variables such as PRKAG1 expression level, age, survival status, clinical stage, WHO grade, and histological diagnosis (**Figure 4A**). Univariate and multivariate COX regression analyses were used to evaluate the correlation between PRKAG1 and HCC prognosis: univariate analysis showed that patient prognosis was significantly associated with PRKAG1 expression (HR=1.038, 95%CI: 1.016–1.060, P<0.01), age (HR=1.020, 95%CI: 1.003–1.037, P<0.01), clinical stage (HR=2.700, 95%CI: 1.466–4.975, P<0.01), T stage (HR=0.219, 95%CI: 0.097–0.494, P<0.01), and M stage (HR=5.340, 95%CI: 1.654–17.339, P<0.01), but not with gender, N stage, height, or weight (**Figure 4B**); multivariate Cox regression analysis showed that PRKAG1 remained an independent prognostic predictor after adjusting for confounding factors such as age, gender, and TNM stage (HR=1.052, 95%CI: 1.010–1.096, P<0.05) (**Figure 4C**). Collectively, these results confirm that PRKAG1 expression level is closely associated with the malignant progression and prognosis of HCC, suggesting that PRKAG1 can serve as an independent prognostic marker for HCC patients.

### 3.4 Co-expression network and functional enrichment analysis of PRKAG1

**Figure 5:**
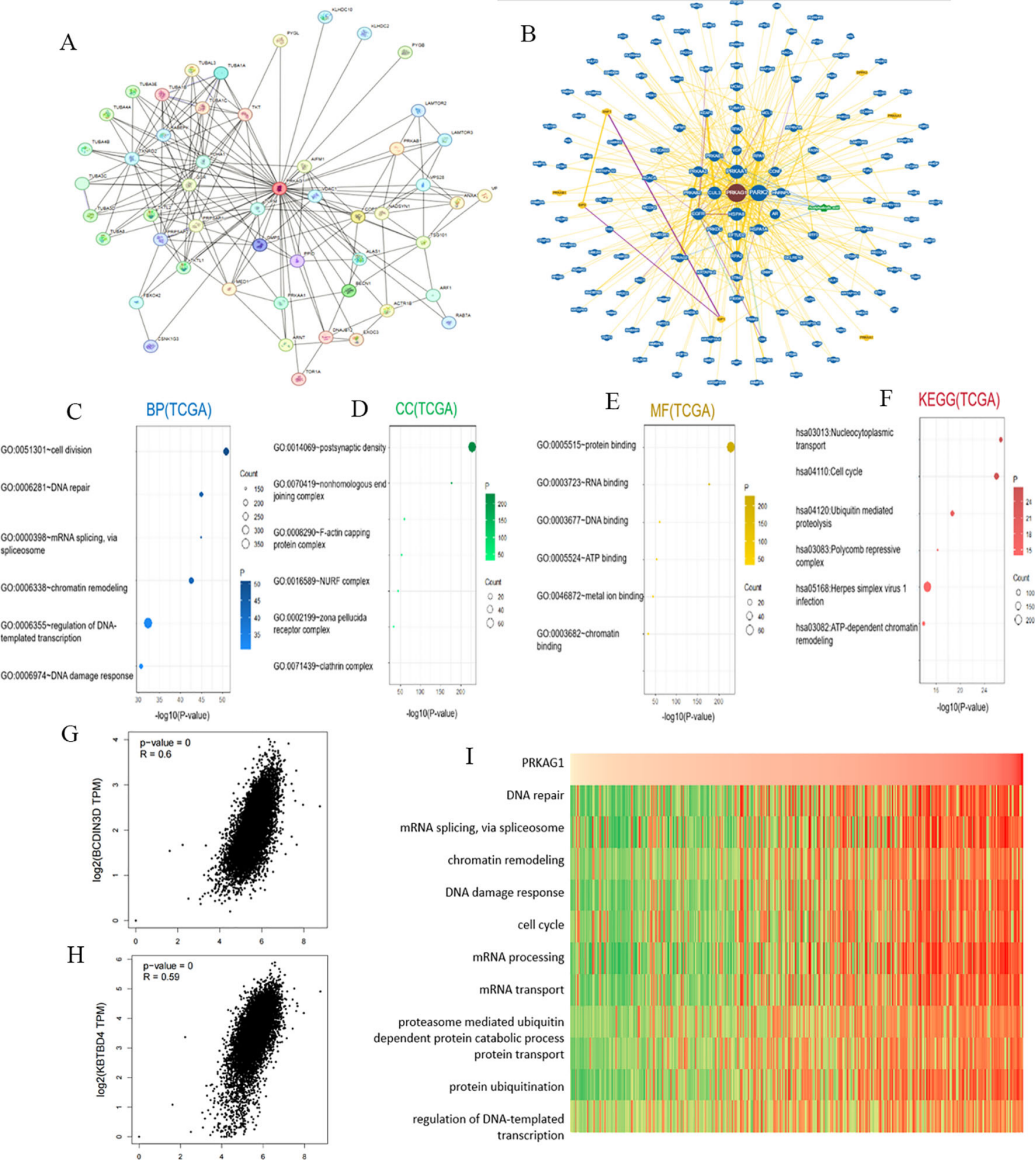
Enrichment Analysis of PRKAG1-Related Genes. **(A)** Co-expression network of 50 genes co-expressed with PRKAG1 obtained through the STRING tool. **(B)** PRKAG1-protein interactions obtained through BioGRID. **(C-F)** Gene Ontology (GO) analysis, including Biological Processes (BP), Cellular Components (CC), Molecular Functions (MF), and Kyoto Encyclopedia of Genes and Genomes (KEGG) pathway analysis, of the top 50 genes co-expressed with PRKAG1 obtained through STRING. **(G, H)** Correlation analysis between PRKAG1 and BCDIN3D and KBTBD4 in all tumor samples from TCGA conducted through GEPIA 2. **(I)** Correlation analysis between PRKAG1 expression and enrichment scores of biological functions, with a heatmap displaying the PRKAG1 expression and enrichment scores of biological functions for each patient in the Cancer Genome Atlas (TCGA) database, samples sorted in ascending order of PRKAG1 expression.

To clarify the molecular mechanism of PRKAG1 in HCC, we constructed a co-expression network using the STRING tool and screened 50 genes with the most similar expression patterns to PRKAG1 (**Figure 5A**). Analysis of the BioGRID4.3 database further revealed direct interacting proteins of PRKAG1, including BCDIN3D, KBTBD4, and CUL3 (**Figure 5B**). Correlation analysis via GEPIA2 showed that PRKAG1 was significantly positively correlated with BCDIN3D (R=0.60, P<0.001) and KBTBD4 (R=0.59, P<0.01) (**Figures 5G, H**), suggesting potential synergistic effects of these proteins in HCC.

GO/KEGG enrichment analysis of the 50 co-expressed genes showed that PRKAG1 is mainly involved in the following biological processes (**Figures 5C–E**): biological process (BP): cell division (P=2.06E-55), DNA repair (P=4.74E-49), and mRNA splicing (P=5.38E-49); cellular component (CC): primarily localized to the postsynaptic density (P<0.01); molecular function (MF): protein binding and RNA binding (P=1.09E-233); KEGG pathway analysis further indicated that PRKAG1-related genes were significantly enriched in nucleocytoplasmic transport (P=6.31E-30), cell cycle regulation (P=5.90E-29), and ubiquitin-proteasome degradation pathways (P=1.68E-21) (**Figure 5F**), suggesting that it may participate in HCC progression by regulating protein homeostasis and cell proliferation.

Given the known functions of PRKAG1-interacting proteins (e.g., BCDIN3D, KBTBD4, CUL3) in cell cycle regulation (19–21), we evaluated the association between PRKAG1 expression and cancer-related pathways using gene set variation analysis (GSVA). Results showed that cell cycle (e.g., G2/M checkpoint) and mitosis-related pathways were significantly activated in the high PRKAG1 expression group (**Figure 5I**), implying that PRKAG1 may drive HCC proliferation by promoting cell cycle progression.

**Figure 6:**
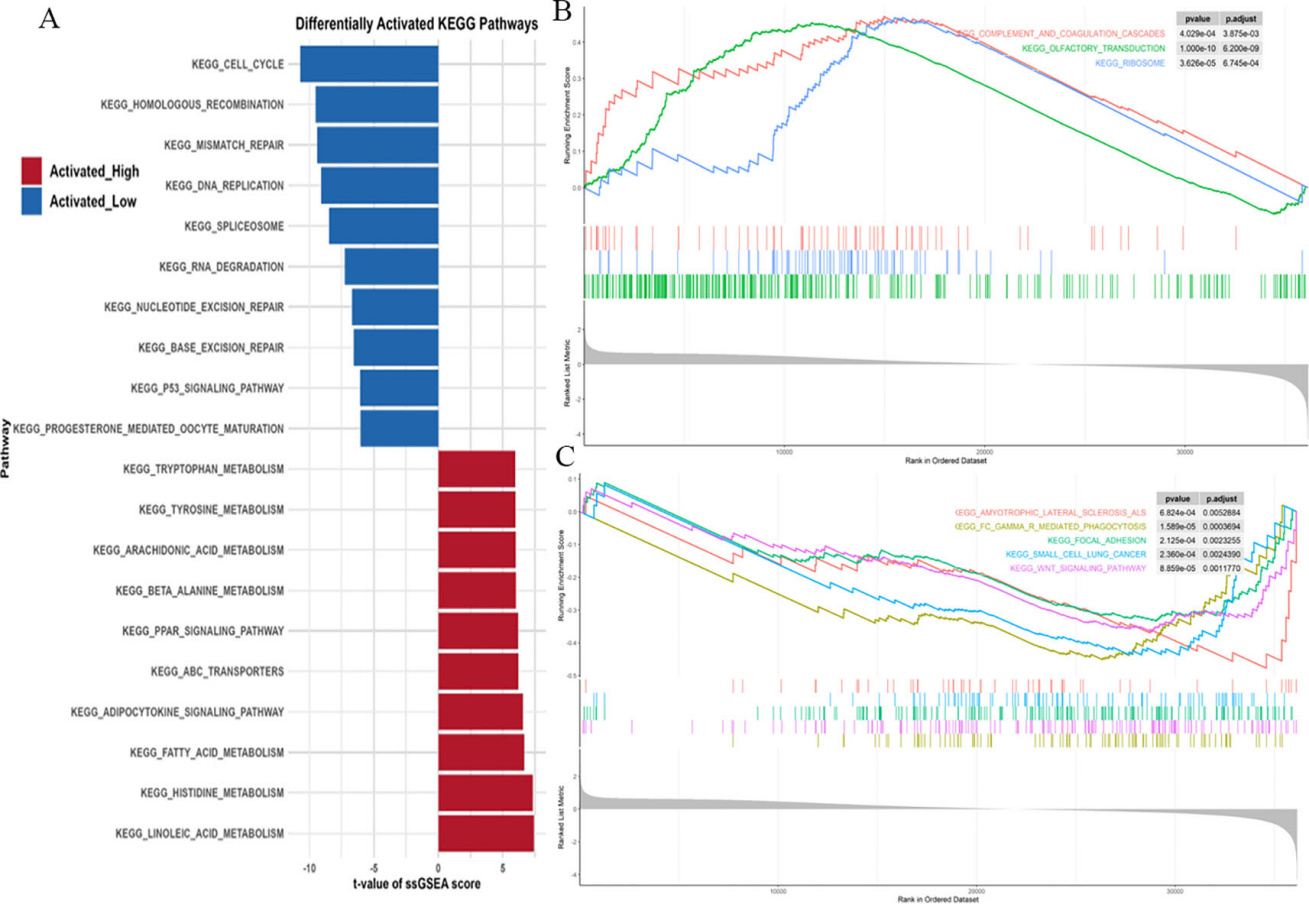
ssGSEA Reveals Distinct KEGG Pathways in PRKAG1 High/Low Expression and Risk Groups. **(A)** ssGSEA analysis of KEGG pathway activity differences between PRKAG1 high- and low-expression groups (MSigDB c2.cp.kegg.v7.4 gene sets). Bar plots display the top 10 significantly enriched pathways in each group (Wilcoxon rank-sum test, log2FC>0.1, P.adj<0.05). **(B, C)** Differential expression analysis by DESeq2, with GSEA revealing top 10 significantly enriched pathways in high-risk vs low-risk groups (FDR<0.25, ranked by |NES|, visualized using clusterProfiler).

Using single-sample gene set enrichment analysis (ssGSEA), we systematically compared KEGG pathway activities between the high and low PRKAG1 expression groups (**Figure 6A**). Analysis based on the MSigDB c2.cp.kegg.v7.4 gene set showed significant differences in pathway activities between the two groups (Wilcoxon rank-sum test, log2FC>0.1, adjusted P<0.05). The top 10 enriched pathways in the high-expression group were analyzed via DESeq2, followed by GSEA to compare pathway enrichment between high- and low-risk groups (**Figure 6B**). The high-risk group showed activation of complement and coagulation cascades, olfactory transduction, and ribosome pathways (FDR<0.25, ranked by |NES|), while the low-risk group was enriched in amyotrophic lateral sclerosis, FcγR-mediated phagocytosis, and focal adhesion pathways (**Figure 6C**). These results were visualized using clusterProfiler, highlighting unique pathway characteristics of different risk groups. Multi-omics analysis indicated that PRKAG1 interacts with key cell cycle regulators and participates in HCC progression through multiple signaling pathways.

### 3.5 PRKAG1 shapes the immune microenvironment through differential immune infiltration and immune-stromal interactions

**Figure 7:**
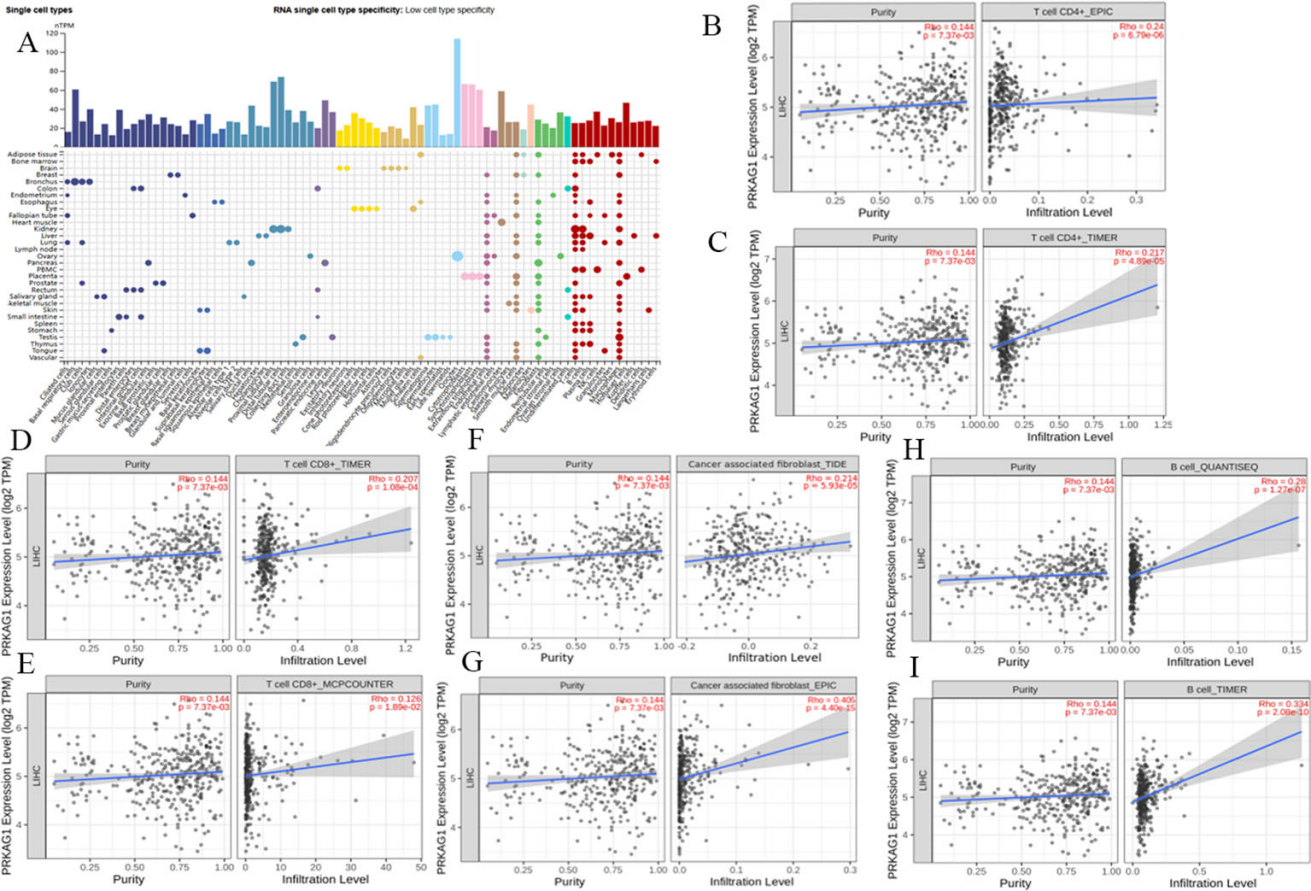
Correlation between PRKAG1 Expression and Immune Infiltration. **(A)** Correlation of PRKAG1 with various tissues and cell types in the HPA database. **(B-I)** Correlation between PRKAG1 expression and immune infiltration of CD4+ T cells, CD8+ T cells, B cells, and cancer-associated fibroblasts in HCC from TCGA, calculated using algorithms such as EPIC, TIMER, TIDE, and MCPCOUNTER. **(J)** Pearson correlation between PRKAG1 and inhibitory immune checkpoints. The width of the bands represents the R-value. The color of the bands represents the P-value.

Given the close association between the tumor immune microenvironment and carcinogenesis, analysis of the HPA database showed significant differences in PRKAG1 expression across different tissues and cell types. Notably, PRKAG1 exhibited relatively high expression levels in multiple immune cell populations (**Figure 7A**). By integrating transcriptome data from TCGA liver cancer samples, we systematically evaluated the correlation between PRKAG1 expression and immune cell infiltration in the tumor microenvironment using multiple algorithms (EPIC, TIMER, TIDE, and MCPCOUNTER). Results showed that PRKAG1 expression was positively correlated with the infiltration of CD4+ T cells, CD8+ T cells, B cells, and cancer-associated fibroblasts (**Figures 7B–I**).

**Figure 8:**
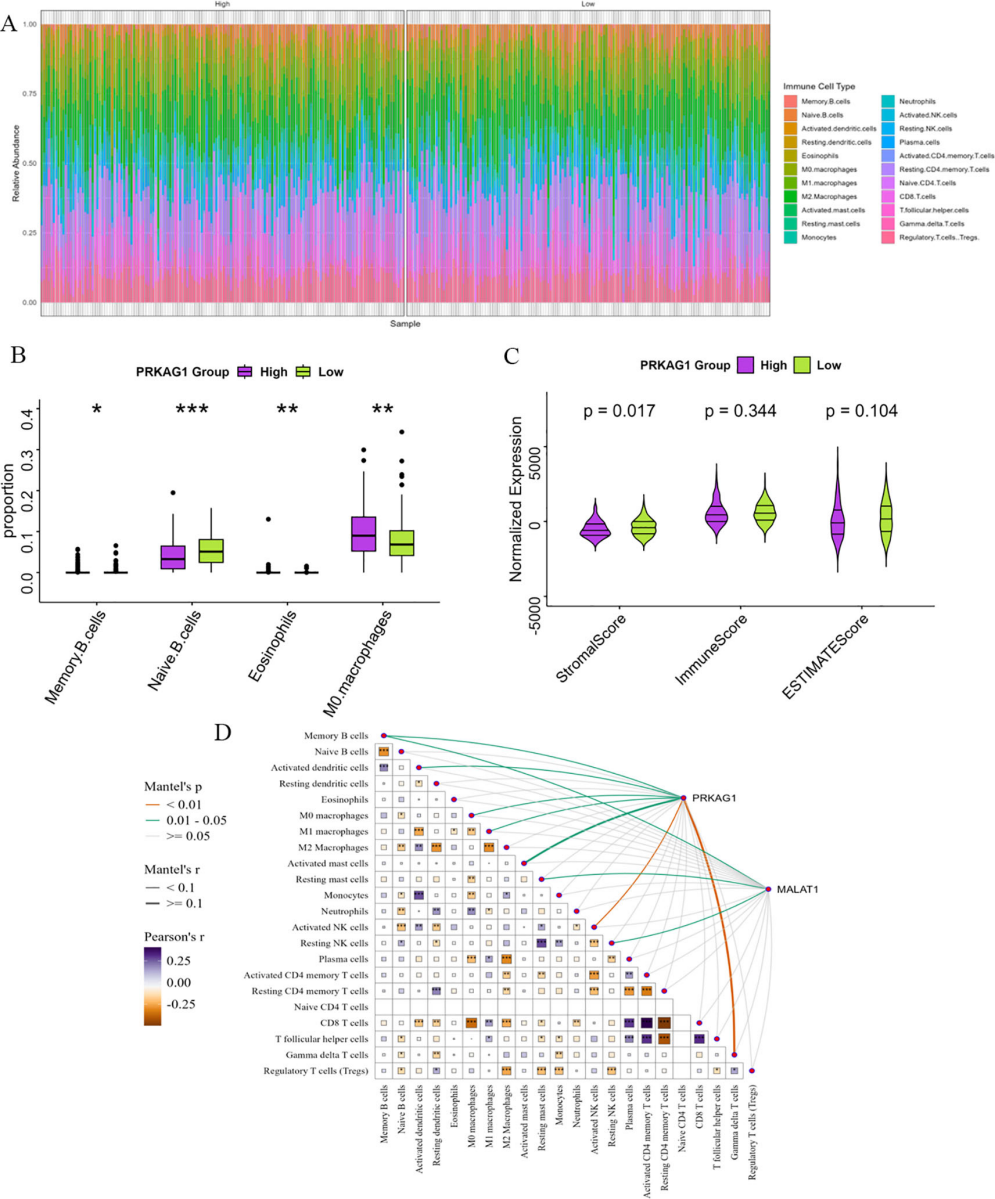
Enrichment Analysis of PRKAG1-Related Genes. **(A)** Patients were divided by median PRKAG1 expression into high/low groups, with CIBERSORT assessing differential immune infiltration. **(B)** The box plots display the differential abundance of memory B cells, naïve B cells, M0 macrophages, and eosinophils (analyzed by CIBERSORT) in PRKAG1-high (red) versus PRKAG1-low (blue) groups. **(C)** Immune and stromal scores were computed using ESTIMATE algorithm for PRKAG1 high/low groups. **(D)** Spearman analysis demonstrated correlations of PRKAG1 and MALAT1 expression with immune cell abundance, visualized via scatter plots with regression lines (95% CI) (*p < 0.05; **p < 0.01; ***p < 0.001; ****p < 0.0001).

HCC patients were divided into high-expression (PRKAG1-high) and low-expression (PRKAG1-low) groups based on the median PRKAG1 expression level. CIBERSORT analysis showed that PRKAG1 expression significantly affected the composition and abundance of immune cells in the tumor microenvironment (**Figure 8A**). Comparative analysis of immune cell subsets between the high (red) and low (blue) PRKAG1 expression groups revealed: significantly reduced proportions of memory B cells (P<0.05) and naive B cells (P<0.001) in the high-expression group; significantly increased infiltration of M0 macrophages (P<0.01) and eosinophils (P<0.01) in the high-expression group (**Figure 8B**). ESTIMATE algorithm analysis showed that the stromal score of the high PRKAG1 expression group was significantly reduced (P<0.05) (**Figure 8C**), indicating that PRKAG1 may participate in the remodeling of the tumor microenvironment by regulating immune-stromal interactions. Spearman correlation analysis further revealed the synergistic regulatory effect of PRKAG1 and MALAT1 expression on immune cell abundance (**Figure 8D**). These findings suggest that PRKAG1 may reshape the HCC immune microenvironment by differentially regulating the infiltration of specific immune cell subsets (e.g., memory B cells, M0 macrophages) and through synergistic effects with MALAT1, thereby influencing tumor immune escape and clinical outcomes.

### 3.6 Single-cell expression characteristics and intercellular communication analysis of PRKAG1

**Figure 9:**
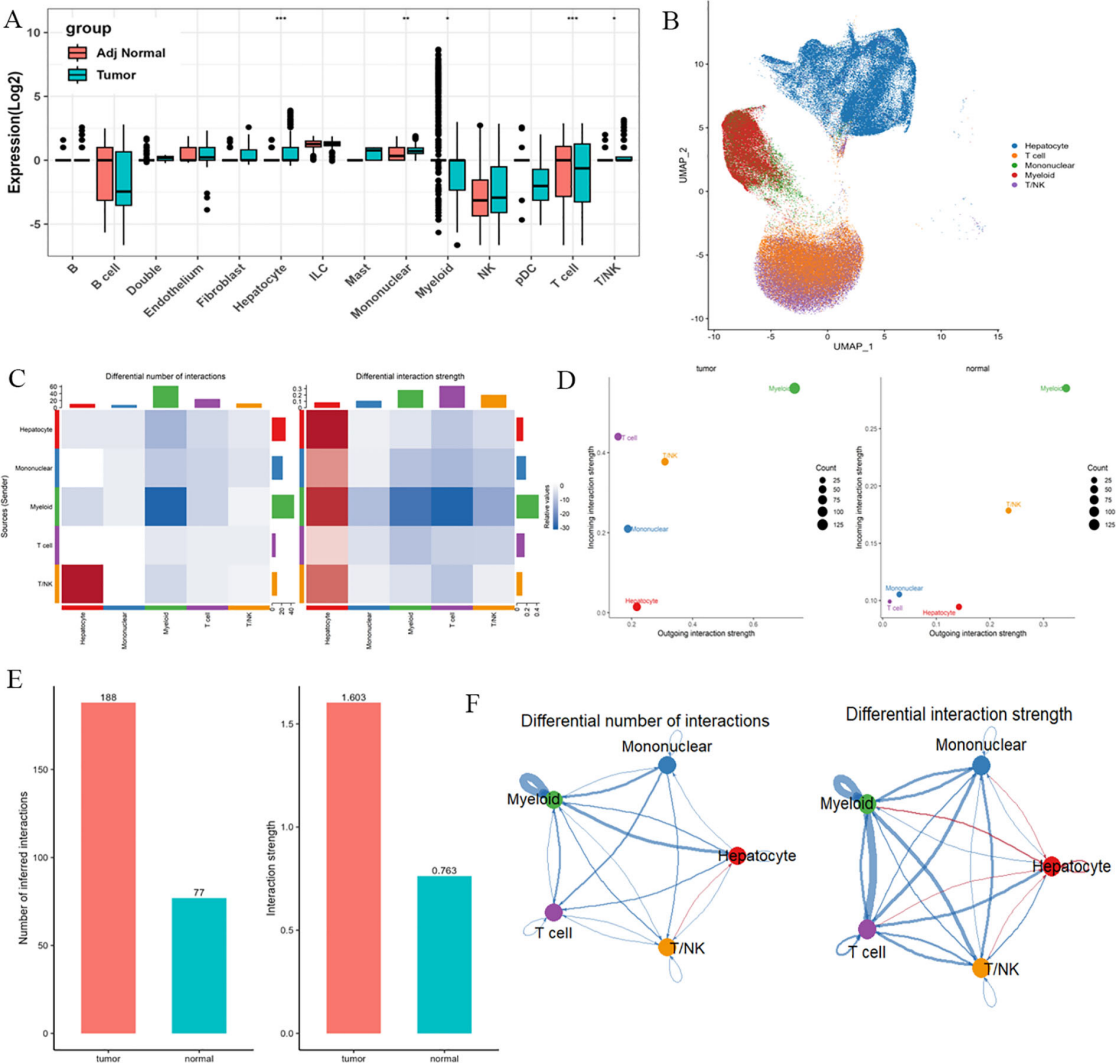
Single-cell expression patterns of PRKAG1 in tumor vs. adjacent tissues and cell-cell communication analysis. **(A, B)** Differential expression analysis of PRKAG1 across cell types between tumor and adjacent normal tissues. **(C, D)** Cell-cell communication analysis evaluating the correlation between PRKAG1 expression and functional signaling strength in hepatocytes, monocytes, myeloid cells, T cells, and T/NK cells. **(E)** Systematic characterization of PRKAG1-expressing cell-specific communication networks in tumor and non-tumor microenvironments using CellPhoneDB algorithm. **(F)** UMAP visualization of monocyte subpopulations, annotated with PRKAG1 expression patterns.

Using single-cell RNA sequencing technology, we systematically analyzed the differential expression patterns of PRKAG1 in various cells from tumor tissues and adjacent normal tissues. Results showed that in the tumor microenvironment, PRKAG1 was significantly highly expressed in hepatocytes and monocytes (P<0.05); in adjacent normal tissues, PRKAG1 was mainly enriched in T cells and myeloid cells (P<0.05) (**Figures 9A, B**). Subsequent intercellular communication analysis found that high PRKAG1 expression in hepatocytes, monocytes, myeloid cells, T cells, and T/NK cells was significantly positively correlated with the signal intensity of multiple pathways (**Figures 9C, D**). Systematic analysis using the CellPhoneDB algorithm showed that PRKAG1-expressing cells were more likely to establish communication networks in the tumor microenvironment (**Figure 9E**). UMAP visualization of monocyte subsets revealed that specific subsets exhibited significant PRKAG1 high-expression characteristics (**Figure 9F**).

**Figure 10:**
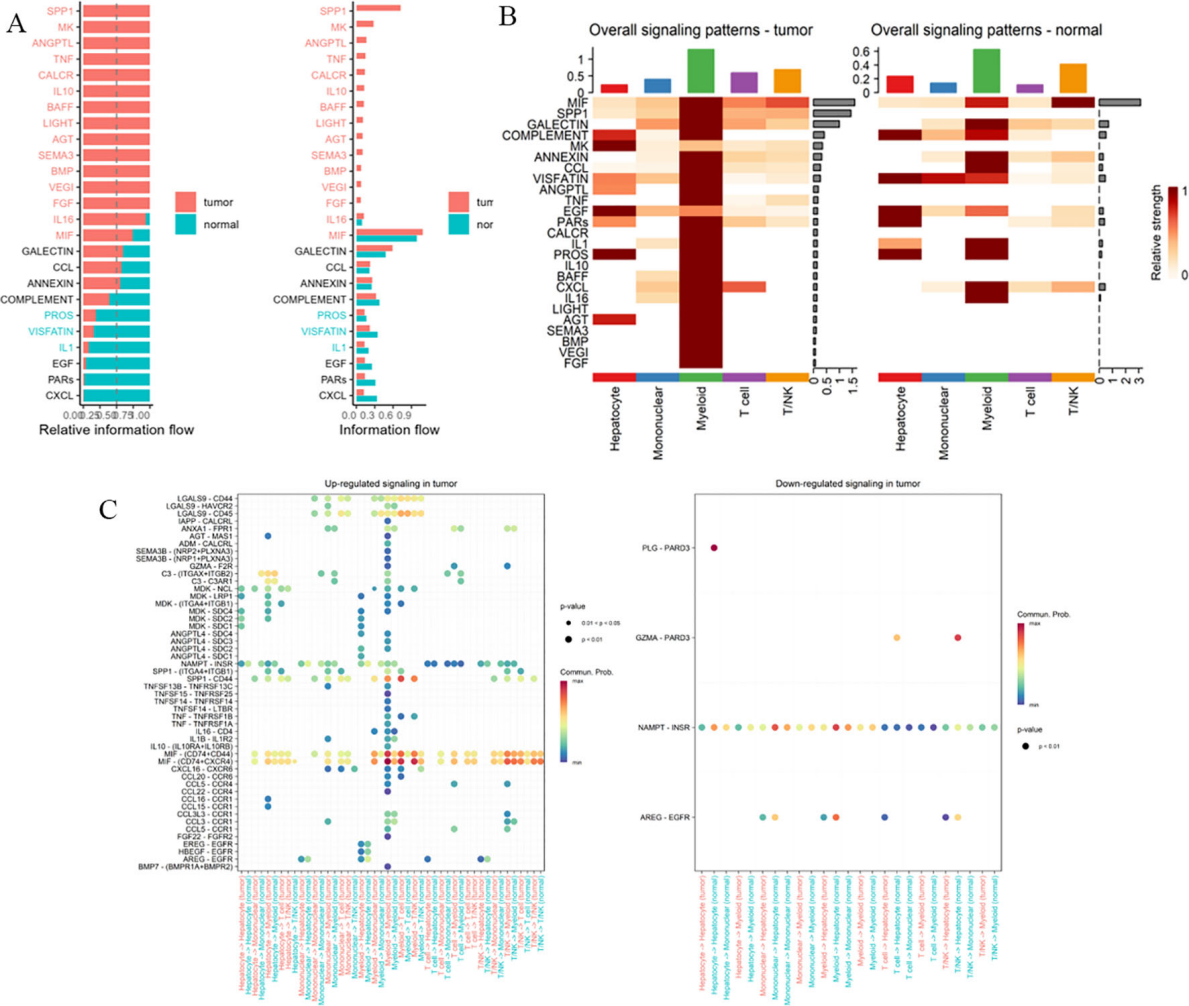
Cell-cell communication network analysis. **(A, B)** Signaling strength of intercellular communication factors between tumor and normal tissues. **(C, D)** Communication signal intensity of cellular factors across different cell types. **(E, F)** CellChat package (v1.6.0) analysis of communication networks between PRKAG1+ monocytes and other immune cells. CellChat integrates a database of known ligand-receptor pairs and quantifies intercellular signaling strength through probabilistic modeling.

Comparative analysis of intercellular communication characteristics between tumor and normal tissues found: significantly enhanced communication intensity of factors such as SPP1 and MIF in tumor tissues; more active communication networks involving SPP1 and lectins in normal tissues (**Figures 10A, B**). Further analysis of signal intensity of communication factors between different cell types showed that hepatocytes and myeloid cells established close communication networks through multiple factors (**Figures 10C, D**). Analysis of the communication network of PRKAG1+ cell subsets using CellChat software (v1.6.0) found that PRKAG1+ myeloid cells interacted with other cells mainly through MIF-(CD74+CD44) and MIF-(CD74+CXCR4) networks, significantly enhancing pathway signal transduction (P<0.01). Probability model quantification showed that the ligand-receptor interaction intensity between PRKAG1+ myeloid cells and other cells increased by 3–5 fold (**Figures 10E, F**). These findings reveal the cell-type-specific expression pattern of PRKAG1 in the tumor microenvironment and its potential mechanism in HCC development by regulating intercellular communication networks.

### 3.7 PRKAG1 promotes proliferation, migration, and invasion of hepatocellular carcinoma cells

**Figure 11:**
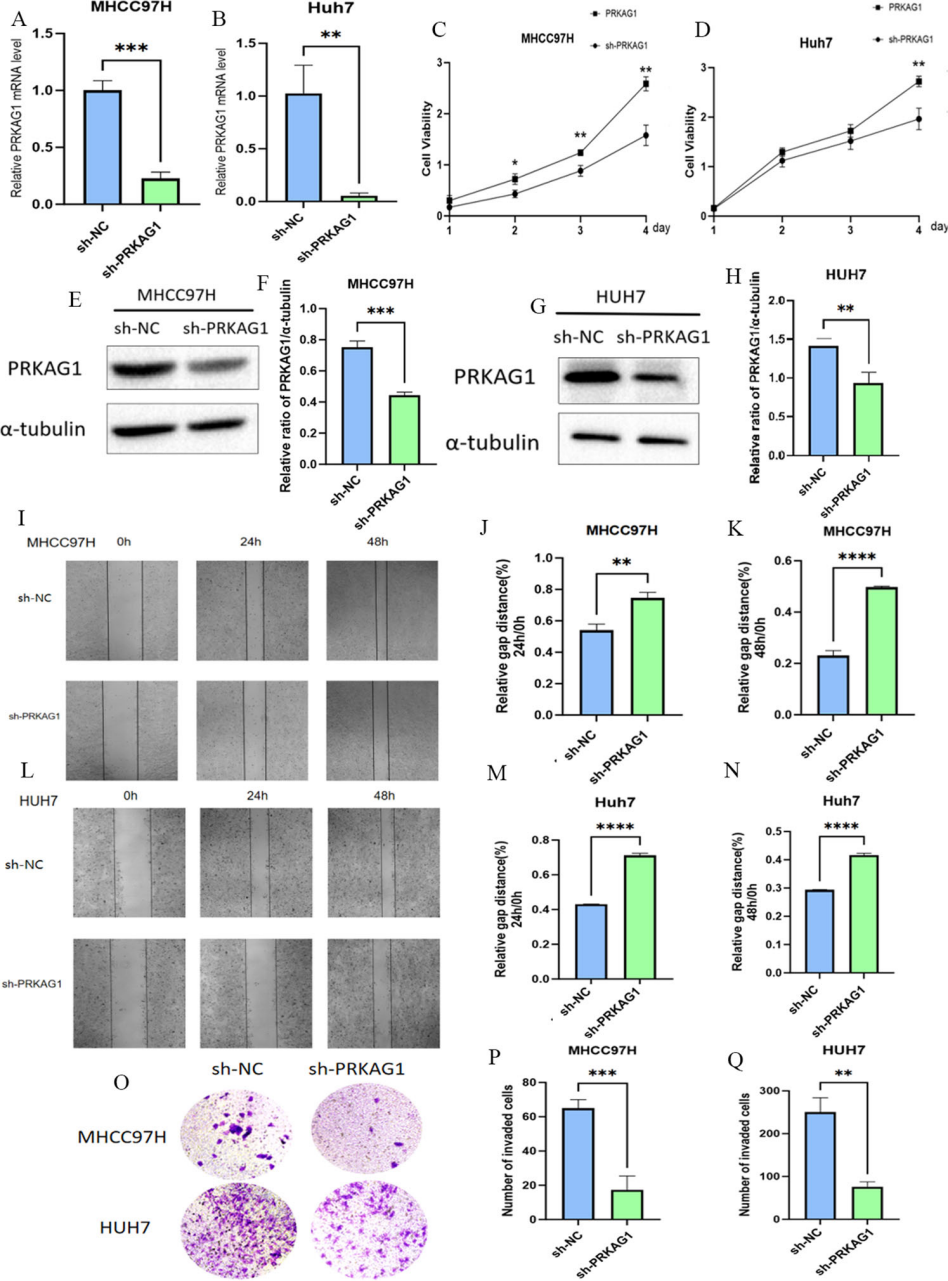
The Role of PRKAG1 in Liver Cancer Cells. **(A, B)** Q-PCR was used to detect PRKAG1 expression in MHCC97H and Huh-7 cells 48 hours after transfection with sh-PRKAG1 or sh-NC. **(C, D)** CCK8 assay was used to detect the proliferation rates of MHCC97H and Huh-7 cells at 24, 48, 72, and 96 hours post-transfection. **(E-H)** Western blot analysis of the transfection efficiency of PRKAG1. **(I-N)** Wound healing assays were performed on MHCC97H and Huh-7 cells transfected with h-PRKAG1 or sh-NC at 24 and 48 hours. **(O-Q)** Transwell assay was used to detect the invasion ability of hepatocellular carcinoma cells after sh-PRKAG1 transfection. Data are presented as mean ± SD. **p < 0.01; ***p < 0.001; ****p < 0.0001 compared to the specified group.

To investigate the role of PRKAG1 in the malignant biological behavior of HCC cells, we first established stable PRKAG1 knockdown models in MHCC97H and Huh-7 cell lines via shRNA transfection. Western blot analysis verified the knockdown efficiency (**Figures 11A, B, E–H**). Functional experiments showed that compared to the negative control group (sh-NC), the proliferation ability of sh-PRKAG1 group cells was significantly reduced at 48–96 hours in CCK-8 assays (**Figures 11C, D**). Wound healing assays indicated that PRKAG1 knockdown weakened cell migration ability (**Figures 11I–N**), while Transwell-Matrigel invasion assays further showed that PRKAG1 deficiency reduced invasive ability by approximately 50% (**Figures 11O–Q**). These results collectively confirm that PRKAG1 plays a critical role in HCC progression by regulating cancer cell proliferation, migration, and invasion, suggesting its significant potential as a therapeutic target.

### 3.8 *In vivo* experiments

**Figure 12:**
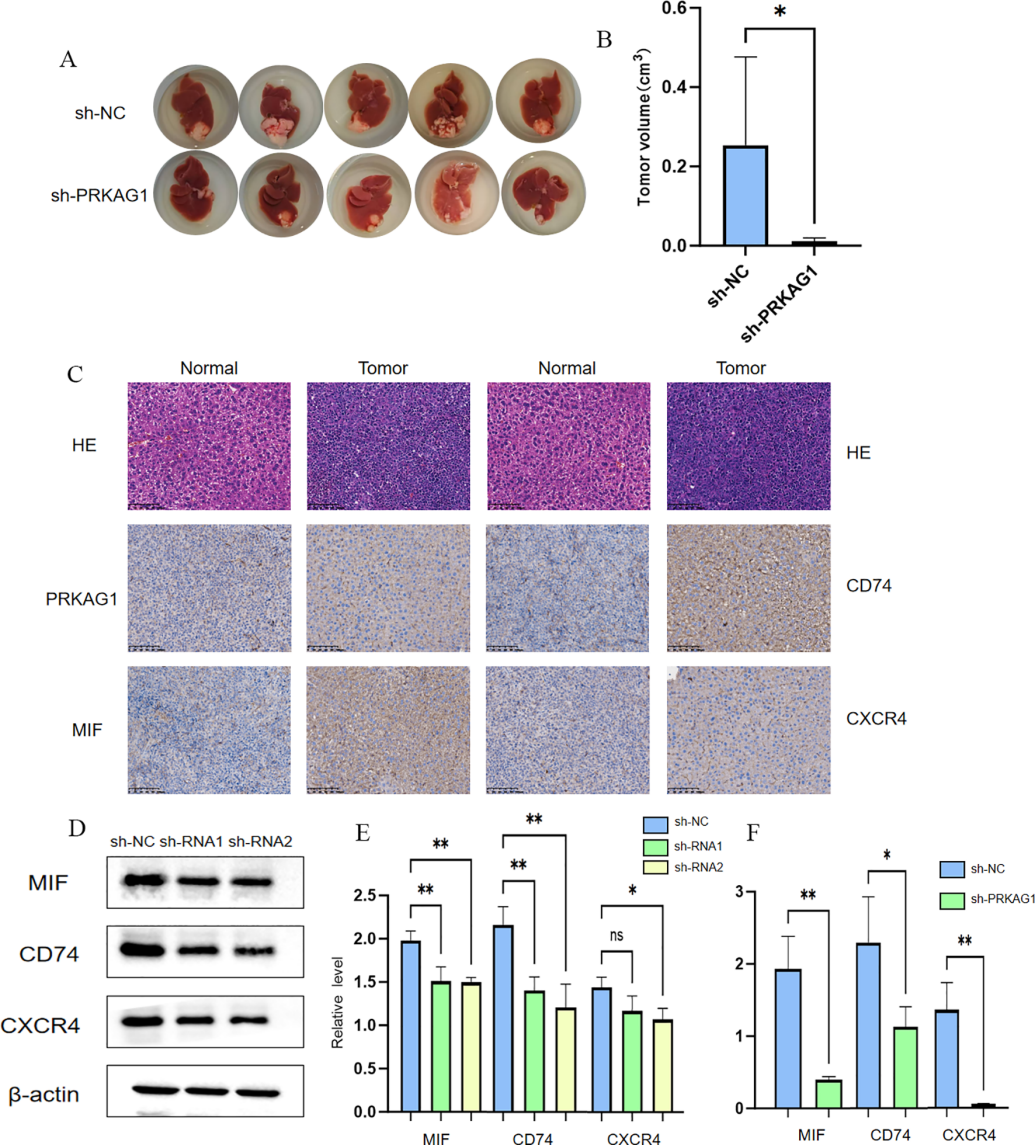
Knockdown of PRKAG1 inhibits xenograft tumor growth. MALAT1 regulates PRKAGI expression bu modulating miR-383-5p. **(A, B)** TARGETSCAN prediction of miR-383-5p binding to MALT1 and PRKAG1. **(C)** RNAhybrid-predicted binding sites in PRKAG1 3'-UTR. **(D)** qRT-PCR of miR-383-5p in normal and HCC cells **(E, F)** miR-383-5p expression after MALAT1 knockdown. **(G-L)** PRKAG1 mRNA and protein levels after MALAT1 knockdown and miR-383-5p inhibition. **(M)** Western blot of P53 and p-AKt. Data are mean ± SD. *p < 0.05.

To further clarify the biological role of PRKAG1 in HCC occurrence and development, this study constructed a mouse orthotopic xenograft model. Mice were divided into the Hepa1-6-shPRKAG1 group (PRKAG1 knockdown group) and the Hepa1-6-shNC group (negative control group). After a 4-week observation period, systematic analysis of tumor formation in both groups was performed. Results showed that the tumor size and volume in the Hepa1-6-shPRKAG1 group were significantly smaller than those in the Hepa1-6-shNC group, intuitively indicating that PRKAG1 knockdown could effectively inhibit the growth of HCC xenografts (**Figures 12A, B**). To verify the pathological characteristics of xenografts and PRKAG1 expression in tumor tissues, HE staining and immunohistochemistry were performed. HE staining results clearly showed the tumor formation status of tumor tissues in both groups, further confirming the successful construction of the orthotopic xenograft model (**Figure 12C**). Meanwhile, immunohistochemical results showed high PRKAG1 expression in tumor tissues, providing an important basis for subsequent exploration of PRKAG1’s mechanism of action (**Figure 12C**).

Previous studies found through comparative analysis of intercellular communication characteristics between tumor and normal tissues that the communication intensity of factors such as MIF in tumor tissues was significantly enhanced, and myeloid cells mainly interacted with other cells through the MIF-(CD74+CXCR4) network, significantly enhancing pathway signal transduction. Based on this, this study verified the expression of MIF, CD74, and CXCR4 in HCC tissues via immunohistochemistry. Results showed that the immunohistochemical scores of MIF, CD74, and CXCR4 in HCC tissues were significantly higher, suggesting that these three molecules may play important roles in HCC occurrence and development (**Figure 12C**). Given that MIF, CD74, and CXCR4 are important molecules and receptors in immune cells, this study further explored the effect of PRKAG1 on the expression of these three molecules. Western blot and RT-qPCR assays showed that the protein and mRNA expression levels of MIF, CD74, and CXCR4 were significantly reduced in the Hepa1-6-shPRKAG1 group with PRKAG1 knockdown (**Figures 12D–F**). These results collectively indicate a close and important relationship between PRKAG1 and the tumor immune microenvironment, and it may influence HCC progression by regulating the MIF-(CD74+CXCR4) signaling pathway.

### 3.9 Molecular mechanism of the MALAT1/miR-383-5p/PRKAG1 regulatory axis

**Figure 13:**
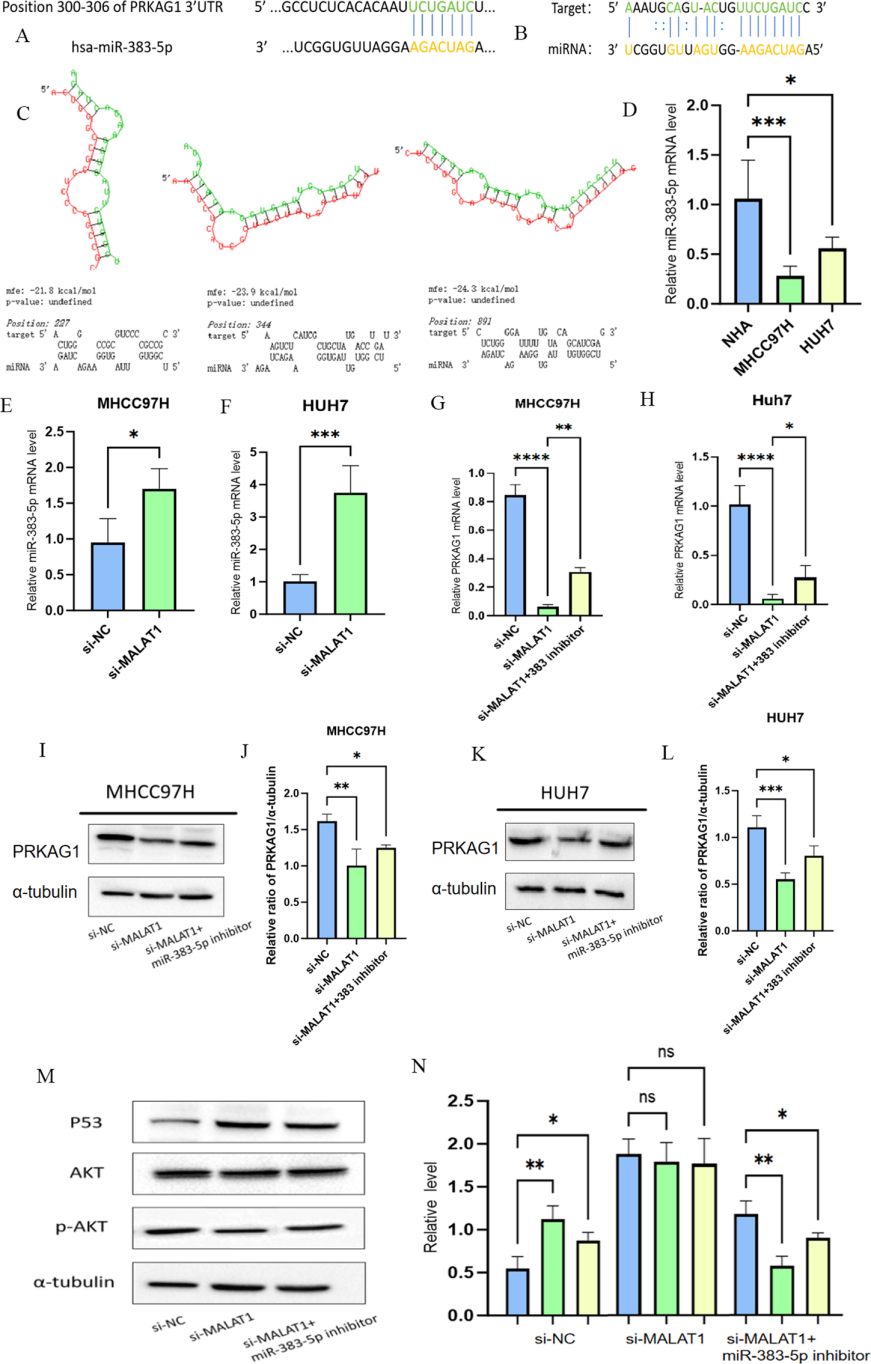
MALAT1 regulates PRKAGI expression bu modulating miR-383-5p. **(A, B)** TARGETSCAN prediction of miR-383-5p binding to MALT1 and PRKAG1. **(C)** RNAhybrid-predicted binding sites in PRKAG1 3'-UTR. **(D)** qRT-PCR of miR-383-5p in normal and HCC cells **(E, F)** miR-383-5p expression after MALAT1 knockdown. **(G-L)** PRKAG1 mRNA and protein levels after MALAT1 knockdown and miR-383-5p inhibition. **(M, N)** Western blot of P53 and p-AKt. Data are mean ± SD. *p < 0.05; **p < 0.01; ***p < 0.001; ****p < 0.0001.

Based on the known regulatory role of the long non-coding RNA MALAT1 in HCC, we further analyzed its molecular pathway regulating PRKAG1 expression through the competing endogenous RNA (ceRNA) mechanism, combining bioinformatics prediction with experimental verification. Systematic analysis via the TARGETSCAN database identified miR-383-5p as a common target miRNA of MALAT1 and PRKAG1 (**Figures 13A, B**). To verify this regulatory relationship, the RNAhybrid tool was used to accurately predict the interaction between the PRKAG1 3’-UTR and miR-383-5p, revealing three high-affinity binding sites (positions 227, 344, and 891) in the 5’ region of the PRKAG1 3’-UTR, and corresponding RNA secondary structure models were successfully constructed (**Figure 13C**). Quantitative real-time PCR (qRT-PCR) analysis showed that miR-383-5p expression was significantly downregulated in MHCC97H and Huh-7 cells compared to normal human hepatocytes (**Figure 13D**). Additionally, after transfection with siMALAT1, miR-383-5p levels in both cell lines were significantly increased compared to the negative control (siNC) (**Figures 13E, F**). Subsequent detection of MALAT1 knockdown on PRKAG1 expression revealed that MALAT1 depletion in MHCC97H and Huh-7 cells resulted in decreased PRKAG1 mRNA and protein levels, which could be significantly reversed by miR-383-5p inhibition (**Figures 13G–L**). These results confirm that MALAT1 upregulates PRKAG1 expression by sponging miR-383-5p through a ceRNA mechanism.

As a key tumor suppressor gene, p53 plays a central role in regulating cell cycle progression, inducing programmed cell death, and maintaining genomic stability (22). Meanwhile, AKT, as a critical effector molecule in the PI3K/AKT/mTOR signaling pathway, exerts serine/threonine kinase activity through phosphorylation activation (p-AKT) to regulate downstream biological processes (23). Our experimental data showed that MALAT1 knockdown significantly reduced the protein expression levels of p53 and p-AKT in MHCC97H and Huh-7 cells, without affecting total AKT abundance. Notably, inhibition of miR-383-5p effectively reversed the downregulation of p53 and p-AKT induced by MALAT1 knockdown (**Figures 13M, N**). These findings confirm the ceRNA function of MALAT1 and its mechanism of influencing downstream signaling molecules through regulating miR-383-5p.

## 4 Discussion

Long non-coding RNAs (lncRNAs) play critical regulatory roles in malignant tumors and are closely associated with the progression of hepatocellular carcinoma (HCC) (24). Previous studies have identified multiple lncRNAs such as HULC, SRA1, and DGCR5 as key drivers of HCC initiation and development (25–27), and dysregulated lncRNA expression is a hallmark feature of HCC. MALAT1 exerts pleiotropic regulatory functions in HCC: it regulates pre-mRNA splicing by interacting with PTBP1 and PSF, modulates SR protein activity, and promotes nucleocytoplasmic shuttling to drive HCC progression. Additionally, it interacts with nuclear speckle components (e.g., SON protein) to regulate the phosphorylation status of SR family splicing factors, thereby influencing alternative splicing patterns in tumor cells (28). Furthermore, MALAT1 enhances vesicle transport and exosome secretion in HCC cells to facilitate oncogenic signal transduction (29); it synergizes with HULC to regulate TRF2 expression and promote the growth of HCC stem cells, while its back-spliced product circ-MALAT1 promotes stem cell self-renewal by inhibiting PAX5 translation (30). Simultaneously, MALAT1 inhibits AXIN1 and APC to activate the Wnt pathway, accelerating cell proliferation (31), highlighting its complex regulatory network.

As a competing endogenous RNA (ceRNA), lncRNAs often regulate mRNA expression by competitively binding miRNAs, and ceRNA networks serve as key regulatory axes in HCC development (32). This study is the first to confirm that MALAT1 upregulates PRKAG1 expression by sponging miR-383-5p to promote HCC, consistent with the tumor-suppressive role of miR-383-5p (6). The function of MALAT1 extends beyond traditional models; its nuclear localization enables participation in multi-dimensional regulation: it forms R-loops to anchor chromatin TAD boundaries and regulate oncogene cluster expression (33); it recruits EZH2 to mediate H3K27 trimethylation and silence tumor suppressor genes (34). This dual regulatory mode provides a novel mechanistic explanation for epigenetic abnormalities in HCC.

PRKAG1, as the γ1 regulatory subunit of AMPK, classically functions to sense cellular energy status (AMP/ATP ratio) and regulate metabolic homeostasis (35). This study is the first to systematically clarify its oncogenic function and prognostic value in HCC, revealing that its abnormally high expression is strongly associated with clinical stage and survival prognosis, suggesting that it may participate in tumor progression through non-classical pathways beyond the classical metabolic regulatory framework. Functional enrichment analysis showed that PRKAG1 is significantly involved in core biological processes such as cell division and DNA repair, and is closely associated with the cell cycle and ubiquitin-proteasome pathways. This highly aligns with the recent research focus on “metabolic-epigenetic crosstalk”—AMPK family members have been confirmed to directly regulate the proliferation of tumor cells by phosphorylating histone acetyltransferases or DNA methyltransferases (36). The downregulation of p53 expression and reduced AKT phosphorylation levels caused by PRKAG1 knockdown reveal that it may exert oncogenic functions through “metabolic sensing-signal transduction” cross-regulation.

Immune remodeling of the tumor microenvironment is a core mechanism underlying immune escape and malignant progression in HCC. To analyze the regulation of immune phenotypes by metabolic molecules, single-cell analysis showed that PRKAG1 is specifically highly expressed in hepatocytes and monocytes in tumor tissues, and its expression pattern is significantly correlated with immune cell infiltration characteristics, suggesting that it may be a key molecule linking intrinsic tumor metabolism and the extrinsic immune microenvironment. Specifically, the reduction in memory B cells and increased infiltration of M0 macrophages in the high PRKAG1 expression group indicate metabolite-mediated remodeling of immune cell function.

More importantly, the discovery that PRKAG1^+^ myeloid cells enhance intercellular communication through the MIF-(CD74^+^CXCR4) pathway establishes a functional association between metabolic molecules and immune checkpoint pathways. MIF, as a key immune regulatory factor, can inhibit T cell activity by binding to CD74/CXCR4 (37), and this study confirmed that PRKAG1 knockdown significantly reduces the expression of MIF, CD74, and CXCR4, suggesting that it may reshape the immunosuppressive microenvironment by regulating the “metabolite-cytokine-receptor” axis. This “metabolic-immune” cross-regulation provides experimental evidence for the “metabolic checkpoint” theory and highlights the dual expression characteristics of PRKAG1 in immune cells and tumor cells.

Although this study reveals the role of the MALAT1/miR-383-5p/PRKAG1 axis in HCC, limitations remain. First, conclusions are mainly based on cellular experiments, with limited validation in animal models. Second, the detailed regulatory network between MALAT1 and PRKAG1 in HCC requires further exploration. Additionally, the precise mechanism by which PRKAG1 influences the tumor immune microenvironment needs further investigation. Future studies should focus on clarifying the clinical significance of MALAT1 and PRKAG1 in HCC and exploring their potential in immunotherapy.

## 5 Conclusion

Through multi-omics analysis and functional validation, this study reveals the core role of the MALAT1/miR-383-5p/PRKAG1 axis in HCC progression and establishes cross-boundary associations between epigenetic regulation, metabolic reprogramming, and immune microenvironment remodeling. This discovery not only expands our understanding of the molecular mechanisms of HCC but also proposes a new framework for combined strategies involving “RNA targeting + metabolic regulation + immunotherapy”. It is expected to open new avenues for precise diagnosis and effective treatment of HCC.

## Data Availability

The raw data supporting the conclusions of this article will be made available by the authors, without undue reservation.
